# Predicting Strict Trifecta Outcomes after Robot-Assisted Partial Nephrectomy: Comparison of RENAL, PADUA, and C-Index Scores

**DOI:** 10.15586/jkcvhl.v8i4.183

**Published:** 2021-10-01

**Authors:** Kaan Karamık, Yasin Aktaş, Ahmet Gürkan Erdemir, Ekrem İslamoğlu, Mahmut Taha Ölçücü, Çağatay Özsoy, Murat Savaş, Mutlu Ateş

**Affiliations:** 1 Department of Urology, Antalya Training and Research Hospital, University of Health Sciences, Antalya, Turkey;; 2 Department of Radiology, School of Medicine, Hacettepe University, Ankara, Turkey

**Keywords:** C-index, PADUA, partial nephrectomy, RENAL, Trifecta

## Abstract

Nephrometry scores are designed to characterize tumors and stratify the surgical complexity. It remains unclear as to which nephrometry score can accurately predict the surgical outcomes. We aimed to assess the utility of radius, exophytic/endophytic, nearness, anterior/posterior, location (RENAL), preoperative aspects and dimensions used for anatomic classifications (PADUA), and centrality index (C-index) nephrometry scores for predicting the strict Trifecta achievement from a single institution series robotic-assisted partial nephrectomy (RAPN). We retrospectively identified the prospectively maintained robotic surgery database records of 91 patients who underwent RAPN between June 2015 and September 2020 in Antalya Training and Research Hospital. The main outcome of the study was the achievement of strict Trifecta (negative surgical margin, no major urologic complications, warm ischemia time ≤25 min, and ≥85% preservation of estimated glomerular filtration rate). A multivariable analysis was performed to identify the factors of strict Trifecta success. The mean patient age was 55.82 ± 13.37 years with a median clinical tumor size of 3.5 cm (IQR 2.5–4.9). The median RENAL, PADUA, and C-index score were 7(IQR 6–8), 8(IQR 7–10), and 2.01(IQR 1.64–2.72), respectively. A strict Trifecta could be achieved in 54 patients (59.3%). Clinical tumor size (P = 0.011), RENAL risk groups (low:reference; intermediate; P = 0.040; high; P = 0.009), PADUA risk groups (low:reference; intermediate; P = 0.044; high; P = 0.001) and C-index risk groups (low:reference; high; P = 0.015) were the independent predictors of strict Trifecta attainment in the multivariate analysis. None of the nephrometry scores were a superior predictor compared to other nephrometry scores in comparative analysis. RENAL, PADUA, and C-index scores were all independent predictors of a strict Trifecta achievement. Our comprehensive comparison of the three scores identified that none of the nephrometry scores proved to be inferior to others nephrometry scores.

## Introductıon

The incidental detection of small renal masses has increased in the present times, and the cases of renal cell carcinoma (RCC) are more frequently diagnosed due to the widespread use of imaging modalities for unrelated reasons (1). Partial nephrectomy (PN) has become the standard surgical treatment for localized RCC, as it offers a better preservation of renal function and an equivalent oncological outcome compared to radical nephrectomy (2, 3). Open, laparoscopic, and robotic surgical techniques can be implemented for PN. It is noted that robotic-assisted PN (RAPN) is increasingly becoming the approach of choice among patients undergoing PN. RAPN overcome some of the technical difficulties pertaining to the laparoscopic technique, providing easier tumor resection, suturing, and repairing of the renal defect, and a shorter learning curve. Several studies on the topic point out to the fact that the clinical outcomes of RAPN are superior compared to the laparoscopic technique (4).

The main aim of PN should be to completely remove the tumor with minimal complication and minimal decrease in renal function. In order to simplify and standardize the reporting and comparison of the outcomes of PN, Hung et al. have proposed “Trifecta”, adopted from radical prostatectomy (5). Trifecta is defined as a combination of negative surgical margin, minimal renal function decrease, and no urological complication. Different definitions of the component minimal renal function decrease have been reported. Some authors have also reported a warm ischemia time, (WIT) ≤25 min, to be a component of the Trifecta (6, 7). In a study conducted by Sharma et al., Trifecta was defined as WIT <30 min (8). In several studies, the authors have applied strict Trifecta (negative surgical margin, no complications of grade 3 or higher, renal function loss <15%, and WIT≤25 min) to evaluate the renal function preservation more precisely (9, 10).

The nephrometry scores are designed to characterize tumor, facilitate cohort comparisons, and stratify surgical complexity. The radius, exophytic/endophytic, nearness, anterior/posterior, location (RENAL) nephrometry score, preoperative aspects and dimensions used for an anatomic classifications (PADUA) score, and the centrality index (C-index) remain the most known and used systems (11–13). The importance of these nephrometry scores in predicting the perioperative and renal functional outcomes of PN has been demonstrated (14–16).

In this study, we aimed to comparatively assess which of three nephrometry scores corralates best with strict Trifecta achievement in RAPN.

## Material and Methods

### Study design and participants

Following the approval of the Institutional Review Board (approval number = 2020-286), we reviewed the charts of the patients who underwent RAPN for a suspicious renal mass between June 2015 and September 2020 in Antalya Training and Research Hospital. The data were retrospectively noted from a prospectively maintained database. The patients with solitary kidney (n = 2), missing data (n = 4), and a follow-up time shorter than 1 year (n = 40) were excluded from the study. The first 25 cases on the learning curve of the surgeons performing RAPN were also excluded. The final group for the study included 91 patients.

### Nephrometry scores

Before the surgery, all the patients underwent a contrast-enhanced computed tomograph (CT) or magnetic resonance imaging (MRI). RENAL, PADUA, and C-index scores were calculated by a radiologist (AGE) and an urologist (YA) according to the protocols described for these systems (11–13). Both the physicians were blinded to patient characteristics, the clinical outomes, and the results of the other observer’s assessments while they were calculating the nephrometry scores. RENAL scores were categorized into low-complexity (score 4–6), intermediate-complexity (score 7–9), and high complexity (score 10–12). The tumor complexity was stratified as low, intermediate, and high if the calculated PADUA score was 6–7, 8–9, and 10–14, respectively. For the C-index score, tumors seperated into two categories of >2.5 (low complexity) and <2.5 (high complexity).

### Surgical technique

The indication for surgery was elective in all the cases. All 91 RAPN operations were carried out by two surgeons (MS and MA) using the da Vinci XI robotic surgical system (Intuitive Surgical Inc, Sunnyvale, CA, USA). Both transperitoneal and retroperitoneal accesses were utilized. The decision for the surgical approach was taken after assessing the location of the tumor and the surgeons’s preference. The vascular pedicle was dissected and isolated with the help of vascular silicon tapes. The decision to clamp renal hilum was taken during the surgery, based on the tumor characteristics and intraoperative findings. The tumor was identified visually and cut out by a cold scissors with 2–5 mm of the parenchymal margin. The tumor bed was oversewn with 3-0 V-lock, parenchymal sutures were made using the sliding clip technique.

### Clinical assessment

Preoperative demographic data (gender, age, body mass index [BMI], and comorbidities), tumor characteristics (tumor side, clinical tumor size, RENAL, PADUA, and C-index scores), perioperative outcomes (surgical approach, WIT, estimated blood loss [EBL], operation time [OT], and hospitalization), and pathology features (tumor size, histological sub-types, tumor grade, pathological stage, and surgical margin status) were recorded. There were complications in the first 30 days after surgery, which were classified according to Clavien-Dindo system. OT was defined as the time from the placement of trocars to the removal of trocars.

The renal function of the group was evaluated preoperatively and 1 year after operation by serum creatinine levels and estimated glomerular filtration rate (eGFR), calculated by the modification of diet in renal disease (MDRD) formula. Preoperative creatinine levels were measured routinely for 3–7 days before the surgery. Absolute and percent change in eGFR was calculated based on the difference between the preoperative and postoperative up to 12 months.

Strict Trifecta was used for investigating the accomplishment of optimal outcomes of RAPN. The achievement of strict Trifecta was defined as the simultaneous fulfillment of the following factors: negative surgical margin, WIT≤25 min, renal function loss <15%, and no significant perioperative complications (Clavien-Dindo grade ≥3).

### Statistical analysis

The statistical analysis was done using IBM SPSS Statistics for Windows, Version 23.0 (IBM Corp., Armonk, NY). The normality assumptions were controlled by the Shapiro–Wilk test. The descriptive analyses were presented using mean ± SD, median (IQR), or n (%), where appropriate. The categorical data were analyzed by Pearson chi-square and Fisher’s Exact test. Mann–Whitney U test and Student’s t test were used for the analysis of non-normally and normally distributed numerical data, respectively. The paired samples t-test was used for parametric comparison of repeated measurements. Friedman test with Bonferroni correction was used for the non-parametric comparison of parameters measured at different times. The receiver operating characteristic (ROC) curve analysis was applied to determine the optimal cut-off point of RENAL, PADUA, and C-index scores for predicting the achievement of strict Trifecta and area under the curve (AUC); sensitivity and specificity were calculated and reported with 95% confidence intervals. The optimal cut-off point of measurements was determined as the value of the maximum Youden index. Delong’s test was used for comparison of AUC values of nephrometry scores. Univariate and multivariate logistic regression analysis was used to determine the independent risk factors associated with the achievement of strict Trifecta. The variables with P < 0.1 in the univariate analyses were further tested in the multivariate model. Since RENAL, PADUA, and C-index score are highly correlated, a separate regression model was created for each variables. To determine the interobserver reliability between the urologist and radiologist for RENAL, PADUA, and C-index scores, the intraclass correlation coefficient (ICC) was calculated. Odds ratio (OR) with corresponding 95% confidence intervals (95% CIs) was reported. A P-value of less than 0.05 was considered statistically significant.

## Results

The descriptive statistics of the study group are shown in Table 1. The mean patient age was 55.82 ± 13.37 years, with a median clinical tumor size of 3.5 cm ( IQR 2.5–4.9). Most patients in the study were male (70.3%), and operated on in the transperitoneal approach (81.3%). The mean BMI was also noted as 27.13 ± 3.7 kg/m^2^.

**Table 1:** Clinical characteristics of patients.

Variables	Overall	Achieving strict Trifecta	Not achieving strict Trifecta	P-Value
Number of patients	91	54	37	–
Mean age, (years)	55.82 ± 13.37	56.28 ± 12.83	55.16 ± 14.28	0.698
Gender, n (%)				
Male	64(70.3)	38(70.4)	26(70.3)	0.992
Female	27(29.7)	16(29.6)	11(29.7)	
Mean BMI, (kg/m^2^)	27.13 ± 3.7	26.43 ± 3.51	28.16 ± 3.79	**0.027**
Diabetes, n (%)	11(12.1)	7(13)	4(10.8)	0.999
Hypertension, n (%)	27(29.7)	14(25.9)	13(35.1)	0.345
Atherosclerosis, n (%)	15(16.5)	8(14.8)	7(18.9)	0.604
Surgical approach, n (%)				
Transperitoneal	74(81.3)	41(75.9)	33(89.2)	0.111
Retroperitoneal	17(18.7)	13(24.1)	4(10.8)	
Tumor side, n (%)				
Left	52(57.1)	32(59.3)	20(54.1)	0.622
Right	39(42.9)	22(40.7)	17(45.9)	
Median clinical tumor size, (IQR) (cm)	3.5(2.5–4.9)	3(2.3–4.2)	4.6(3.5–5.4)	**<0.001**
Hilar clamping, n (%)				
Total clamp	74(81.3)	38(70.4)	36(97.3)	**0.001**
Off clamp	17(18.7)	16(29.6)	1(2.7)	

BMI, body mass index.

The operative findings and pathological outcomes are summarized in Table 2. Median WIT, OT, and EBL were 24 min (IQR 16–29), 140 min (IQR 120–170), and 100 mL (IQR 60–200), respectively. The median pathological tumor size was 3.5 cm (IQR 2.5–5), and 74.7% (n = 68) of the tumors were RCC, while 25.3% (n = 23) of the tumors were benign. A majority of the tumors were stage T1 (94.1%). Four (5.9%) patients had a positive surgical margin. At a median follow-up of 34 months, no tumor recurrence was reported, and no conversion to open surgery was observed. The overall complication rate was 11% (n = 10) of which only 1.1% (n = 1) was Clavien III. The complications included hemorrhage necessitating blood tranfusion (n = 8), urine leak necessitating JJ ureteral stent placement, and ileus resolved with medical therapy (n = 1).

**Table 2:** Perioperative outcomes and pathological results.

Variables	Overall	Achieving strict Trifecta	Not achieving strict Trifecta	P-Value
Median WIT, (IQR) (min)	24(16–29)	19(0–24)	30(28–32)	**<0.001**
Median OT, (IQR) (min)	140(120–170)	127.5(110–150)	155(140–180)	**<0.001**
Median EBL, (IQR) (mL)	100(60–200)	100(50–200)	150(100–200)	**0.044**
Median pathological tumor size, (IQR) (cm)	3.5(2.5–5)	2.8(2.1–4)	4.5(3.5–5.5)	**<0.001**
Tumor type, n (%)				
Benign	23(25.3)	15(27.8)	8(21.6)	0.507
Malign	68(74.7)	39(72.2)	29(78.4)	
Benign subtype (n = 23), n (%)				
Angiomyolipoma	7(30.4)	4(26.7)	3(37.5)	0.217
Chronic pyelonephritis	2(8.7)	1(6.7)	1(12.5)	
Benign cyst	3(13)	2(13.3)	1(12.5)	
Oncocytoma	6(26.1)	6(40)	0(0)	
Tubulointerstitial nephritis	1(4.3)	0(0)	1(12.5)	
Hydatid cyst	2(8.7)	1(6.7)	1(12.5)	
Ksantogranulomatosis	1(4.3)	1(6.7)	0(0)	
Metanephric adenoma	1(4.3)	0(0)	1(12.5)	
Malign subtype (n = 68), n (%)				
Clear	46(67.6)	23(59)	23(79.3)	0.191
Papillary	15(22.1)	11(28.2)	4(13.8)	
Chromofobe	7(10.3)	5(12.8)	2(6.9)	
Positive surgical margin (n = 68), n (%)	4(5.9)	0(0)	4(13.8)	**0.029**
Pathological stage (n = 68), n (%)				
T1a	38(55.9)	30(76.9)^a^	8(27.6)^b^	**<0.001**
T1b	26(38.2)	9(23.1)^a^	17(58.6)^b^	
T2a	2(2.9)	0(0)^a^	2(6.9)^a^	
T3a	2(2.9)	0(0)^a^	2(6.9)^a^	
WHO/ISUP grade (n = 68), n (%)				
1	14(20.6)	12(30.8)	2(6.9)	0.051
2	42(61.8)	21(53.8)	21(72.4)	
3	11(16.2)	6(15.4)	5(17.2)	
4	1(1.5)	0(0)	1(3.4)	
Complication, n (%)	10(11)	4(7.4)	6(16.2)	0.306
Complication grade (n = 10), n (%)				
1	1(10)	0(0)	1(16.7)	0.999
2	8(80)	4(100)	4(66.7)	
3	1(10)	0(0)	1(16.7)	
Hospitalization, (IQR) (days)	3(3–3)	3(3–3)	3(3–3)	0.375

WIT, warm ischemia time; OT, operation time; EBL, estimated blood loss; WHO/ISUP, World Health Organization/International Society of Urological Pathology.

The association of nephrometry scores and risk groups with strict Trifecta achievement is given in Table 3. The median RENAL, PADUA, and C-index score were 7 (IQR 6–8), 8 (IQR 7–10), and 2.01 (IQR 1.64–2.72), respectively. Strict Trifecta was achieved in patients with a lower RENAL and PADUA score, and a higher C-index score, representing a significant difference (all, P < 0.001).

**Table 3:** Overall nephrometry scores and distrubitions.

	Overall	Achieving strict Trifecta	Not achieving strict Trifecta	P-Value
Median RENAL, (IQR)	7(6–8)	6(5–7)	8(7–9)	**<0.001**
RENAL risk, n (%)				
Low	33(36.3)	28(51.9)^a^	5(13.5)^b^	**<0.001**
Moderate	48(52.7)	24(44.4)^a^	24(64.9)^a^	
High	10(11)	2(3.7)^a^	8(21.6)^b^	
Median PADUA, (IQR)	8(7–10)	8(7–8)	10(9–11)	**<0.001**
PADUA risk, n (%)				
Low	26(28.6)	24(44.4)^a^	2(5.4)^b^	**<0.001**
Moderate	36(39.6)	22(40.7)^a^	14(37.8)^a^	
High	29(31.9)	8(14.8)^a^	21(56.8)^b^	
Median C-index, (IQR)	2.01(1.64–2.72)	2.47(1.96–3.53)	1.66(1.42–1.89)	**<0.001**
C-index risk, n (%)				
Low	31(34.1)	28(51.9)	3(8.1)	**<0.001**
High	60(65.9)	26(48.1)	34(91.9)	

RENAL, Radius, exophytic/endophytic, nearness, anterior/posterior, location; PADUA, Preoperative aspects and dimensions used for an anatomic classifications; C-index, Centrality index.

The renal functional change is demonstrated in Table 4. Median creatinine was 0.95 mg/dL (IQR 0.82–1.08) before the surgery, and it increased to 1.1 mg/dL (IQR 0.92–1.31) on the first postoperative day, and 1.01 mg/dL (IQR 0.84–1.2) 12 months after the surgery. The mean preoperative and postoperative eGFR were 82.68 ± 18.79 mL/min/1.73 m^2^ and 78.25 ± 19.31 mL/min/1.73 m2, respectively. The reduction in eGFR in the first year was statistically significant (P < 0.001).

**Table 4:** Preoperative and postoperative renal functional outcomes.

Variables	Overall	Achieving strict Trifecta	Not achieving strict Trifecta	P-Value
Median preoperative creatinine, (IQR) (mg/dL)	0.95(0.82–1.08)	0.93(0.82–1.07)	0.96(0.83–1.1)	0.513
Median postoperative creatinine, (IQR) (mg/dL)	1.1(0.92–1.31)	1.07(0.92–1.23)	1.12(0.97–1.38)	0.270
Median first year creatinine, (IQR) (mg/dL)	1.01(0.84–1.2)	0.96(0.79–1.12)	1.04(0.87–1.24)	0.131
**P**	**<0.001**	**<0.001**	**<0.001**	
Mean preoperative eGFR, (mL/min/1.73 m^2^)	82.68 ± 18.79	83.24 ± 18.11	81.86 ± 19.96	0.734
Mean first year eGFR, (mL/min/1.73 m^2^)	78.25 ± 19.31	80.8 ± 18.4	74.54 ± 20.25	0.130
**P**	**<0.001**	**0.019**	**<0.001**	
Mean eGFR difference	-4.51 ± 8.42	-2.61 ± 7.36	-7.27 ± 9.17	**0.009**
Mean eGFR percent change	-5.08 ± 10.22	-2.83 ± 9.05	-8.35 ± 11.04	**0.011**

eGFR, estimated glomerular filtration rate.

Table 5 demonsrates the relationship between nephrometry score risk groups and strict Trifecta components. Each scoring system was found to have a statistically significant association with WIT (P = 0.001 for RENAL, P < 0.001 for PADUA and C-index). Additionally, PADUA correlated with renal function reduction (P = 0.001).

**Table 5:** Differences between RENAL, PADUA and C-index score risk groups in terms of strict Trifecta components.

Variables	RENAL				PADUA				C-index		
	Low	Moderate	High	P	Low	Moderate	High	P	Low	High	P
Strict Trifecta achievement, n (%)	28(84.8)^a^	24(50)^b^	2(20)^b^	**<0.001**	24(92.3)^a^	22(61.1)^b^	8(27.6)^c^	**<0.001**	28(90.3)	26(43.3)	**<0.001**
WIT, n (%)											
≤25 min	29(87.9)^a^	26(54.2)^b^	3(30)^b^	**0.001**	25(96.2)^a^	22(61.1)^b^	11(37.9)^b^	**<0.001**	29(93.5)	29(48.3)	**<0.001**
>25 min	4(12.1)^a^	22(45.8)^b^	7(70)^b^		1(3.8)^a^	14(38.9)^b^	18(62.1)^b^		2(6.5)	31(51.7)	
Surgical margin (n = 68), n (%)											
Negative	26(100)	30(88.2)	8(100)	0.166	19(100)	24(92.3)	21(91.3)	0.547	23(100)	41(91.1)	0.292
Positive	0(0)	4(11.8)	0(0)		0(0)	2(7.7)	2(8.7)		0(0)	4(8.9)	
Complication (grade≥3) (n = 10), n (%)											
No	3(100)	4(80)	2(100)	0.999	3(100)	2(66.7)	4(100)	0.600	3(100)	6(85.7)	0.999
Yes	0(0)	1(20)	0(0)		0(0)	1(33.3)	0(0)		0(0)	1(14.3)	
Renal function reduction, n (%)											
≤15%	31(93.9)	42(87.5)	7(70)	0.117	25(96.2)^a^	35(97.2)^a^	20(69)^b^	**0.001**	30(96.8)	50(83.3)	0.090
>15%	2(6.1)	6(12.5)	3(30)		1(3.8)^a^	1(2.8)^a^	9(31)^b^		1(3.2)	10(16.7)	

RENAL, Radius, exophytic/endophytic, nearness, anterior/posterior, location; PADUA, Preoperative aspects and dimensions used for an anatomic classifications; C-index, Centrality index; WIT, warm ischemia time.

Strict Trifecta could be achieved in 54 patients (59.3%). The most common reason for the failure of strict Trifecta was prolonged WIT. Univariate and multivariate analyses were performed to evaluate the preoperative variables predicting strict Trifecta achievement (Table 6). The result of the multivariate analysis showed that the clinical tumor size (OR = 0.596 95% CI = 0.4–0.887; P = 0.011), RENAL risk groups (low:reference; intermediate; OR = 0.278 95% CI = 0.082–0.941; P = 0.040; high; OR = 0.074 95% CI = 0.011–0.521; P = 0.009), PADUA risk groups (low:reference; intermediate; OR = 0.181 95% CI = 0.034–0.958; P = 0.044; high; OR = 0.058 95% CI = 0.01–0.333; P = 0.001), and C-index risk groups (low:reference; high; OR = 0.163 95% CI = 0.038–0.699; P = 0.015) were independent predictors of strict Trifecta success.

**Table 6:** Univariate and multivariate analyses of factors affecting the achievement of strict Trifecta.

Variables	Univariate analysis		Multivariate analysis with RENAL risk		Multivariate analysis with PADUA risk		Multivariate analysis with C-index risk	
	OR (%95 CI)	P	OR (%95 CI)	P	OR (%95 CI)	P	OR (%95 CI)	P
Age	1.006(0.975–1.038)	0.694	**–**	**–**	**–**	**–**	**–**	**–**
Female gender	0.995(0.398–2.486)	0.992	**–**	**–**	**–**	**–**	**–**	**–**
BMI	0.875(0.776–0.988)	**0.031**	0.888(0.775–1.017)	0.086	0.895(0.781–1.026)	0.111	0.89(0.778–1.018)	0.089
Diabetes	1.229(0.333–4.539)	0.757	–	–	–	–	–	–
Hypertension	0.646(0.26–1.603)	0.346	–	–	–	–	–	–
Atherosclerosis	0.745(0.245–2.27)	0.605	–	–	–	–	–	–
Clinical tumor size	0.499(0.347–0.718)	**<0.001**	0.596(0.4–0.887)	**0.011**	0.642(0.435–0.946)	**0.025**	0.674(0.449–1.01)	0.056
Preoperative GFR	1.004(0.982–1.027)	0.730	–	–	–	–	–	–
RENAL score	0.496(0.356–0.691)	**<0.001**	–	–	–	–	–	–
RENAL risk								
Low	Reference	–	Reference	–	–	–	–	–
Moderate	0.179(0.059–0.54)	**0.002**	0.278(0.082–0.941)	**0.040**	–	–	–	–
High	0.045(0.007–0.275)	**0.001**	0.074(0.011–0.521)	**0.009**	–	–	–	–
PADUA score	0.384(0.259–0.57)	**<0.001**	–	–				
PADUA risk								
Low	Reference	–	–	–	Reference	–	–	–
Moderate	0.131(0.027–0.642)	**0.012**	–	–	0.181(0.034–0.958)	**0.044**	–	–
High	0.032(0.006–0.166)	**0.001**	–	–	0.058(0.01–0.333)	**0.001**	–	–
C-index score	7.278(2.818–18.797)	**<0.001**	–	–	–	–	–	–
C-index risk								
Low	Reference	–	–	–	–	–	Reference	–
High	0.082(0.022–0.299)	**<0.001**	–	–	–	–	0.163(0.038–0.699)	**0.015**

RENAL, Radius, exophytic/endophytic, nearness, anterior/posterior, location; PADUA, Preoperative aspects and dimensions used for an anatomic classifications; C-index, Centrality index; BMI, body mass index; eGFR, estimated glomerular filtration rate.

**Figure 1:**
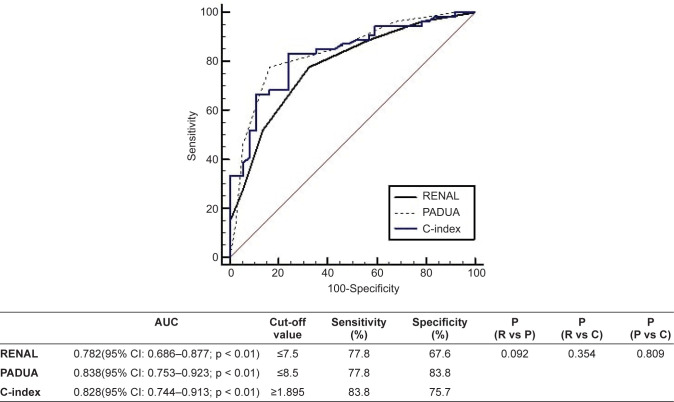
Receiver operating characteristic analysis for RENAL, PADUA, and C-index score to predict the achievement of strict Trifecta.

The ability of the nephrometry scores to predict strict Trifecta outcomes was evaluated by ROC curve analysis (Figure 1). All the scores were good predictors of strict Trifecta (AUCs of RENAL, PADUA, and C-index were 0.782, 0.838, and 0.828, respectively). None of the nephrometry scores were a superior predictor compared to other nephrometry scores.

All three nephrometry scores demonstrated good concordance between the two observers (Table 7). The ICC values between the radiologist and urologist for RENAL, PADUA, and C-index were 0.83, 0.88, and 0.89, respectively.

**Table 7:** Concordance between urologist’s and radiologist’s score using intraclass correlation coefficient.

	ICC	95%CI
**RENAL**		
Radius	0.892	0.841–0.928
Exophytic/endophytic	0.866	0.802–0.910
Nearness	0.730	0.617–0.813
Location	0.759	0.656–0.835
RENAL score	0.832	0.756–0.886
**PADUA**		
Polar location	0.822	0.742–0.879
Exophytic rate	0.876	0.817–0.916
Renal rim	0.825	0.746–0.881
Renal sinus	0.656	0.521–0.759
UCS	0.772	0.674–0.844
Tumor size	0.911	0.867–0.940
PADUA score	0.888	0.834–0.924
**C-index**	0.896	0.847–0.930

RENAL, Radius, exophytic/endophytic, nearness, anterior/posterior, location; PADUA, Preoperative aspects and dimensions used for an anatomic classifications; C-index; UCS, urinary collecting system.

## Discussion

In this study, the ability to predict strict Trifecta for RENAL, PADUA, and C-index scores were evaluated. We observed a good reproducibility of three nephrometry scores among the observers. RENAL, PADUA, and C-index scores were all independent predictor of a strict Trifecta achievement. Our comprehensive comparison of the three scores identified that none of the nephrometry scores proved to be inferior to the others.

The objective of PN is to achieve a satisfactory oncological outcome and minimize complications while preserving renal function to the extend possible. Accordingly, Hung et al. (5) introduced the Trifecta to jointly evaluate the oncological outcomes, renal function preservation, and complications. The Trifecta represents a good tool to asses the success of PN. Currently, there is no consensus on the definitions used for components of the Trifecta. The renal function preservation component of the Trifecta has been interpreted differently by the authors (6–10). The only use of WIT≤25 min does not apply properly to demonstrate long term renal functional outcomes. Thus, we used both: an intraoperative variable that WIT≤25 min and a 85% eGFR preservation at 1 year to define a minimal renal function decrease. However, the main concept of the Trifecta is the same; negative surgical margin, minimal renal function decrease, and safety procedure. In the present study, the positive surgical margin rate of 5.9% is in line with other studies on RAPN. Furthermore, the overall complication rate was 11%, which consistent with previous results (6, 7).

The Trifecta achievement rate in previous studies has ranged between 31.6% and 87.8% (17–19). Several reasons such as surgical experience, different definitions of Trifecta, and surgical approach could have been implicated as adversely influencing the outcomes of Trifecta. Moreover, patient related factors such as age, preoperative eGFR and comorbid status could also affect the renal functional outcomes. The effect of the surgical technique on Trifecta success was investigated, and conflicting results were obtained (20–22). Acar et al. studied 133 patients who underwent open PN or RAPN, and found that the Trifecta rate was similar between the open and robotic techniques (20). Similarly, Mehra et al. compared Trifecta rates after open, laparoscopic, and robotic PN and found no significant differences (21). In contrast, Zargar et al. reported that robotic PNs were more likely than laparoscopic approach to achieve the Trifecta (22). In a study evaluating the surgical experience found that increased experience leads to higher proportion of Trifecta (19). In our study, the strict Trifecta rate was 59.3%. All patients underwent RAPN, and the first 25 cases of both the surgeons were excluded to avoid the effect of the learning curve.

Nephrometry scores have been developed to describe tumor complexity and to standardize the reports of PN. An ideal nephrometry score should have some value in estimating surgical outcomes, and result in consistent scores between observers. In this study, we evaluated the RENAL, PADUA, and C-index scores which are most known and used ones. The correlation between urologist and radiologist seems to be sufficient to recommend the use of RENAL, PADUA, and C-index scores. Likewise, good concordance amongst readers was found in a study on interobserver variability of the RENAL, PADUA, and C-index for robotic and laparoscopic PN patients (8). There are many studies investigating the nephrometry scores and PN outcomes. The external validation studies demonstrated that nephrometry scores associated with perioperative and renal functional outcomes (14–16, 23). However, there are only four studies on comparative analysis of nephrometry scores to predict the Trifecta success (8, 20, 23, 24). In our study, RENAL, PADUA, and C-index scores were all helpful to independently predict the strict Trifecta outcomes. None of the nephrometry scores were superior predictor compared to other nephrometry scores. Egen et al., in a single center study including 150 patients, also support our finding that RENAL, PADUA, and Mayo Adhesive Probability (MAP) scores were independent predictors of the Trifecta (24). Crockett et al. reported the outcomes of 322 RAPNs, and evaluated RENAL, PADUA, and Simplified PADUA REnal classification (SPARE) scores. PADUA and SPARE had superior predictors in terms of Trifecta (23). In another study, the Trifecta outcomes of 50 patients using RENAL, PADUA, and C-index scores had been evaluated. This study revealed that only C-index correlated with the Trifecta achievement. However, there was no univariate and multivarite analyses in this study (8). Contradictory to the above mentioned studies, Acar et al. noted that none of the RENAL, PADUA, and C-index scores showed significant association to predict Trifecta outcomes. On the other hand, the surgeon’s clinical judgement which was evaluated by visual analogue score was more efficient than nephrometry scores to predict Trifecta (25). Although it is subjecitve, this study shows that the clinical opinion and experience of the surgeon who will perform the operation are also important for the favorable outcome.

The predictors of Trifecta has been evaluated by several studies. In a Japanese multicenter study, tumor diameter, EBL and hilar location of the tumor were the independent predictors of Trifecta (7). RENAL score failed to have such a link in the multivariate analysis. Cetindag et al. Published a retrospective case series of laparoscopic PN with 128 patients, and reported that the tumor size was only a predictor of Trifecta (10). In a similar study including 63 patients undergoing laparoscopic PN in T1a renal masses, Osaka et al. reported that the tumor size and surgeon’s learning curve were predictors of Trifecta outcomes (26). Similar to this findings, the experience of the surgeon and the size of the tumor independently predicted Trifecta outcomes (19). Another study reported that the significant predictors of Trifecta success were the tumor size, EBL, OT, robotic surgery, and high risk RENAL scores in the multivariate analysis (22). Harke et al. noted that the PADUA score was only a predictor of Trifecta (27). Conversely, the PADUA score was not associated with Trifecta outcomes (28). In this study, clinical tumor size and nephrometry scores were significant predictors of strict Trifecta. There were inconsistencies in the predictive factors for Trifecta achievement. This can be attributed to the surgical experience, different definitions of Trifecta and surgical approach.

The present study has several limitations. Firstly, our database is prospectively recorded, and the analysis is carried out is retrospective. Secondly, the study involved a limited number of patients within a single center. Finally, RAPNs were performed by 2 surgeons. However, both surgeons have extensive experience with open, laparoscopic, and robotic PNs. Additionally, as we want to minimize the effects of the surgeons’ learning curve of RAPN, we excluded the first 25 patients for both surgeons.

## Conclusion

Based on the results of this study, we suggest using RENAL, PADUA, and C-index scores to predict strict Trifecta outcomes preoperatively, with reproducible interobserver agreement. These scoring systems should undergo an external validation in prospective study with larger study group to predict the Trifecta success.
